# Management of Pipkin Fractures Using a Safe Surgical Hip Dislocation

**DOI:** 10.1155/2019/3526018

**Published:** 2019-10-23

**Authors:** Rita Henriques, Diogo Ramalho, Joaquim Soares do Brito, Pedro Rocha, André Spranger, Paulo Almeida

**Affiliations:** ^1^Hip and Pelvis Unit, Orthopedic Department, University Hospital of Santa Maria, Avenida Professor Egas Moniz, 1649-035 Lisbon, Portugal; ^2^Hip and Pelvis Unit, Orthopedic Department, CUF Descobertas Hospital, Rua Mário Botas (Parque das Nações), 1998-018 Lisbon, Portugal

## Abstract

**Introduction:**

Pipkin fractures are rare events and usually occur as a consequence for high-energy trauma. Surgery to obtain anatomical reduction and fixation is the mainstay treatment for the majority of these injuries; nonetheless, controversy exists regarding the best surgical approach.

**Description of the Case:**

We present the case of a 41-year-old male, which sustained a type II Pipkin fracture following a motorcycle accident. In the emergency department, an emergent closed reduction was performed, followed by surgery five days later. Using a surgical hip dislocation, a successful anatomical reduction and fixation was performed. After three years of follow-up, the patient presented with a normal range of motion, absent signs for avascular necrosis or posttraumatic arthritis, but with a grade II heterotopic ossification.

**Discussion:**

Safe surgical hip dislocation allows full access to the femoral head and acetabulum, without increasing the risk for a femoral head avascular necrosis or posttraumatic arthritis. Simultaneously, this surgical approach gives the opportunity to repair associated acetabular or labral lesions, which explains the growing popularity with this technique.

**Conclusion:**

Although technically demanding, safe surgical hip dislocation represents an excellent option in the reduction and fixation for Pipkin fractures.

## 1. Introduction

Femoral head fractures are severe and uncommon high-energy injuries and can be associated with hip dislocation, fractures of the acetabulum or femoral neck [1–4]. Since the first description of a femoral head fracture associated with hip dislocation in 1869 by Birkett [5], several cases have been reported. In 1957, Pipkin [6] proposed a classification system for these fractures, and as a result, these fractures are more commonly known among the orthopedic community by this eponym (Table 1). Additionally, such injuries have been related to poor functional outcomes and a high rate of complications, particularly avascular necrosis (AVN) and early posttraumatic arthritis [7–9].

It is well established that an early reduction, stabilization, and rigid fixation are essential in order to achieve a stable and congruent articulation, minimizing potential complications [1, 3]. However, there is still controversy regarding the best surgical approach and when to perform a capital fragment fixation or excision [2].

Several surgical approaches have been advocated for the management of these injuries, and all have in common a limited exposure of the femoral head [1, 8–11]. More recently, Ganz et al. [12] described a technique for a safe surgical hip dislocation (SHD), which does not jeopardize femoral head vascularization, allowing in the process full access to the femoral head and acetabulum. Herein, we report the clinical case of a patient with a type II Pipkin fracture treated using a SHD. This work has been reported in line with the SCARE criteria [13].

## 2. Description of the Case

**Figure 1:**
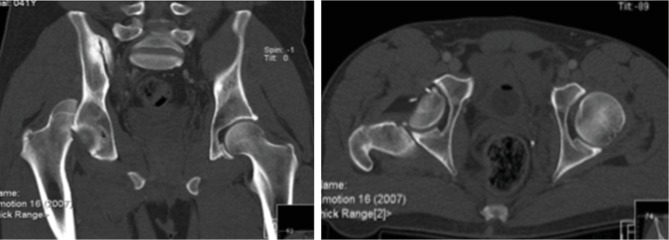
CT-scan of the pelvis showing a femoral head fracture associated with a posterior hip dislocation.

**Figure 2:**
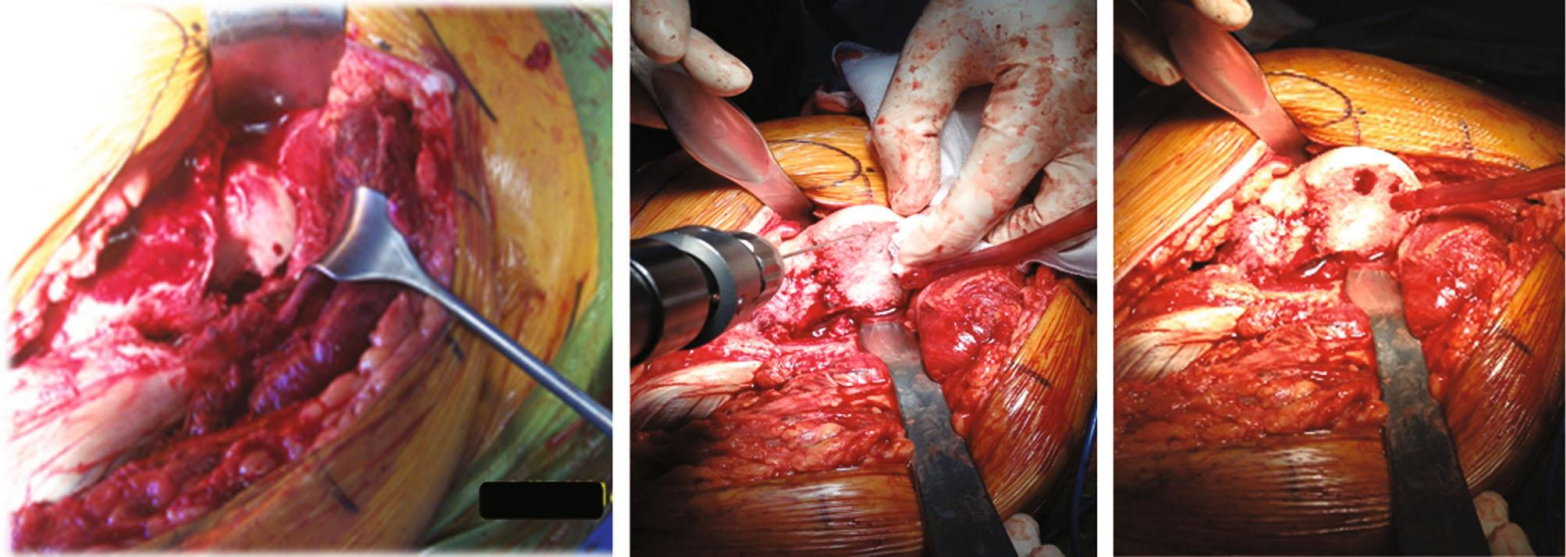
Intraoperative exposure of the femoral head through a surgical hip dislocation approach and intraoperative assessment of femoral head vascularization performed with K-wire perforation.

**Figure 3:**
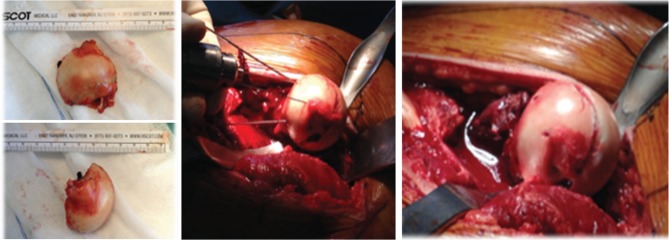
Femoral head fragment and its rigid fixation with 3 subchondral cannulated screws.

**Figure 4:**
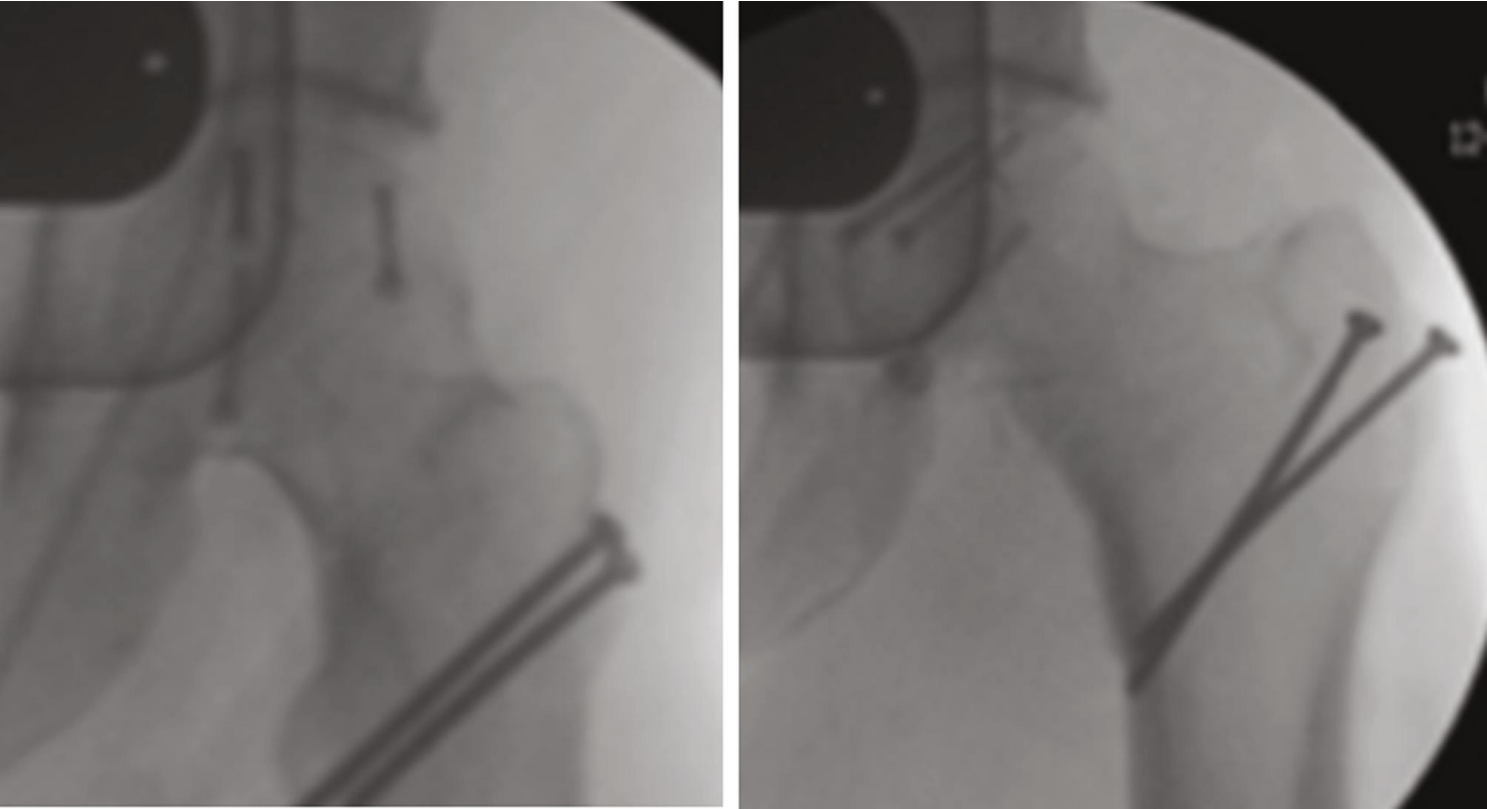
Trochanteric fixation with two 3.5 mm cortical screws.

A 41-year-old male sustained a posterior hip dislocation with an associated type II Pipkin fracture following a motorcycle accident (Figure 1). In the emergency department, an emergent close reduction under fluoroscopy was performed, followed by definitive surgical treatment five days after the initial injury. This delay in the definitive orthopedic treatment was due to a concomitant aortic rupture, which needed an emergent vascular repair. Regarding the Pipkin fracture, we chose to perform an anatomical reduction and internal fixation (Figure 2), using a surgical hip dislocation as described by Ganz et al. [12]. The labrum was also inspected, and a posteroinferior lesion was identified and repaired. We additionally verified the viability for the femoral head, performing perforations with a small K-wire as preconized in literature [12] (Figure 2). The osteosynthesis was achieved using three subchondral headless cannulated screws (Figure 3), the capsule was closed with Vicryl 2-0 sutures, and the greater trochanter stabilized using two 3.5 mm cortical screws (Figure 4).

**Figure 5:**
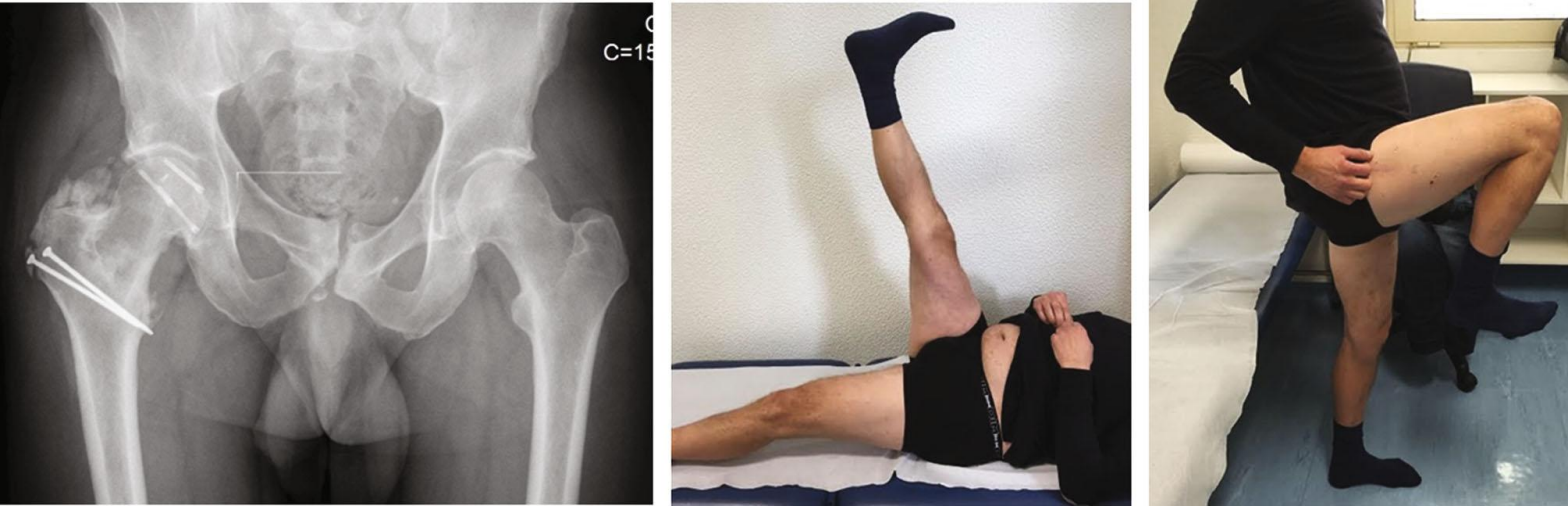
Simple anteroposterior radiograph showing supratrochanteric heterotopic ossification at 3 years of follow-up, with a good active motion of the hip and painless hip flexion.

Postoperatively, the patient started partial weight-bearing on the operated limb at 10 weeks after the index surgery. Five months later, the patient was able to walk without crutches and painless range of motion (Figure 5). He maintained a regular follow-up in the outpatient clinic, and after three years, a full range of motion was still present, without any signs of avascular necrosis or significant degenerative joint disease. Nonetheless, a Brooker grade II heterotopic ossification could be identified in the radiographs (Figure 5).

## 3. Discussion

The surgical treatment for Pipkin fractures remains a source of controversy, especially regarding the best surgical approach [1, 9, 12]. These fractures are often associated with hip dislocations and therefore is imperative to recognize it and promote prompt reduction in order to decrease the risk for AVN [2, 3, 12]. The incidence of AVN in hip dislocations ranges from 8 to 26% [2], with reduction below six hours after the injury as a benchmark to restore vascular supply to the femoral head [3].

Butler in 1981 [14] described a case series comparing closed reduction versus operative treatment in Pipkin type II fractures and showed that if the fracture fragment was anatomically reduced by closed means, it was not necessary to promote its surgical fixation. However, this treatment has been almost abandoned due to the high rate of complications related to longstanding patient immobility [3]. Nowadays, most femoral head fractures are treated surgically, since it is known that an anatomic reduction is associated with a better functional outcome [3, 9]. In cases of Pipkin type I and II fractures, the free capital fragments should be fixed and stabilized whenever possible, in order to decrease the odds of progression to an early degenerative hip [2]. However, not all femoral head fractures are prone to osteosynthesis. The dichotomy between simple excision and internal fixation is clearly dependent on the size of fragments, degree of comminution, and anatomical location, especially regarding the femoral head loading surface area [2, 3]. Nonetheless, literature shows consistently better results with fixation in comparison to fragment excision [15].

Traditionally, the most common surgical approaches for femoral head fixation are posterior (Kocher-Langenbeck), anterior (Smith-Peterson), and anterolateral (Watson-Jones). However, all of them have limited exposure of the femoral head and acetabulum, which makes an anatomical reduction and identification of associated injuries inside the acetabulum or in the labrum difficult [1, 3]. In older studies, Epstein and colleagues [7] strongly recommended fixation of femoral head fractures through a posterior approach. They postulated that the anterior approach was associated with an increased rate of AVN, since it could disrupt the blood flow through the ascending lateral femoral circumflex artery and cause additional damage to the vascular supply of the femoral head [7, 12]. It is now recognized that lateral femoral circumflex artery does not constitute the main source of vascularization of the femoral head [3]. In 1992, Swiontkowski et al. [11] found that the anterior Smith-Peterson approach was a superior to posterior approach concerning blood loss and surgical time and had no increased incidence of avascular necrosis. However, it was related to a higher rate of heterotopic ossification [11, 16].

Several recent cadaveric studies have shown that the medial femoral circumflex artery (MFCA) is the main source for femoral head vascularization, contributing for the main intra- and extracapsular anastomotic rings, and it enters the capsule superolaterally. Surgeons must be aware of these anatomic features since this branch can be at a particular risk in posterior approaches [17, 18].

SHD, described by Ganz et al. [12], is performed through a trochanteric-flip osteotomy, avoiding in this way disinserting external hip rotators, which contribute to the protection of MFCA, the most important branch in femoral head vascularization. The capsulotomy, usually Z-shaped, allows to rise a retinacular flap, which contributes also to preserve local blood supply [12]. Intraoperatively, it is recommended to check the vascularization of the femoral head, which can be tested through small head perforations. A bleeding sign correlates positively with femoral head viability [10, 12]. This assessment can also be performed using a laser Doppler flowmetry as preconized by Nötzli et al. [19]. SHD additionally allows full access to the entire femoral head and acetabulum, promoting an anatomical reduction of the capital fragments, and the identification of chondral, subchondral, or labral injuries that could go unnoticed using other approaches [1, 2, 12]. According to some series, labrum lesions occur in up to 50% of these patients and its presence is related to a worse functional outcome [2].

In order to achieve a proper fixation, we can use subchondral headless screws, countersinking lag screws, bioabsorbable pins, or screws/suture fixation [2, 3, 20]. In this setting, the use of synthetic biodegradable implants can show some advantages as they are easily manipulated and require no further removal. Although the good results, some foreign body reactions have been documented with these implants in some locations [20]. Otherwise, metal implants can lead to stress shielding, allergic reactions, or even early and late infections [21]. The use of osteochondral autologous graft transfer (OATS) remains as another surgical possibility. First described by Hangody and Karpati in the 1990s for the treatment of focal chondral and osteochondral lesions of the knee and talus, it remains also a valid technique, especially when cartilage defects are present within femoral weight-bearing surface [7, 20, 22]. The donor site can be the lateral femoral condyle [20] or the non-weight-bearing intact surface of the ipsilateral femoral head. This technique has demonstrated good autograft incorporation and favorable clinical midterm outcomes [20, 22].

Major complications as heterotopic ossification, AVN, and posttraumatic arthritis can strongly affect long-term results for these patients [15]. It is not clearly defined if heterotopic ossification relates with the surgical approach [4, 9], and despite some authors recommend systematic use of indomethacin as prophylaxis, there are no clear guidelines to its prevention [1, 4]. A large systematic review published by Giannoudis et al. [23] reported a higher incidence of heterotopic ossification using a trochanteric-flip osteotomy (47.2%), when comparing with anterior (44.7%) or posterior (32.3%) surgical approaches. Mostafa et al. [4] also reported a high heterotopic ossification incidence (33.3%) in patients with Pipkin type I and II fractures treated exclusively by a trochanteric-flip approach. Although the exact pathogenesis is still unknown, several factors have been associated with the development of ectopic bone, including polytrauma, craniocerebral or thoracoabdominal trauma, male sex, associated acetabular fractures, mechanical ventilation, delay to surgery, and femoral head injuries [24].

Stannard et al. [8] showed that the Kocher-Langenbeck approach was associated with a 3.2 times higher incidence of AVN compared with the anterior approach. This trend was also confirmed by Giannoudis et al. systematic review [23]. AVN rate in Pipkin fractures treated using a SHD rounds 7.7 to 11.8%. However, it must be stressed that most actual series still have a short follow-up period, which precludes a proper evaluation of the AVN rates [25]. Nonetheless, in 2015, Gavaskar and Tummala [2] reported a series of 26 patients with Pipkin fractures submitted to SHD and they found no cases of AVN with a mean follow-up of 36 months.

Trochanteric-flip osteotomy showed a lower progression rate to posttraumatic arthritis comparatively to anterior or posterior approaches [23, 25]. In this setting, Giannoudis et al. [23] reported a 30 and 20 times higher rate of posttraumatic arthritis when a posterior or an anterior approach was used, respectively, comparing to a trochanteric-flip approach. On the other hand, Gavaskar et al. reported an 11.5% posttraumatic arthritis rate using SHD [2]. However, nonunion of the trochanteric osteotomy is a potential complication for the SHD approach only [4, 12].

Scolaro et al. [26] reported a large series of Pipkin fractures and concluded that Pipkin type III fractures have consistently worse results than type I or II, with a higher failure of fixation and AVN rate, which often leads to a hip arthroplasty conversion. Instead, most of Pipkin type II fractures are amenable to fixation (88%) union without significant complications [26]. Prognosis for Pipkin type IV fractures is mainly determined by the type and severity of concomitant acetabular fractures [27].

When comparing patient functional outcomes, recent literature shows consistently satisfactory functional results in patients submitted to femoral head fixation through a trochanteric-flip approach, making it a safe and optimal choice for the operative treatment of these fractures [2, 12, 23, 25]. Gavaskar and Tummala recorded in their prospective trial a mean modified Merle d'Aubigne score of 16.53 points, at a mean follow-up of 36 months. These outcomes were classified as excellent in 9, good in 15, and fair in 2 patients. For the same series, the mean Oxford hip score was 42.65 points [2]. In other study by Mostafa et al., the Thompson-Epstein and modified Merle d'Aubigne scoring systems were used. At an average of 31 months of follow-up, ten out of twelve patients (83.3%) treated for Pipkin type I and II fractures using a trochanteric-flip osteotomy showed good to excellent results [4].

Finally, we should be aware that the outcomes of these patients are dependent on a wide variety of factors such as the severity of the initial injury and other concomitant injuries, patient health status, timing until initial reduction and surgery [4], and postoperative complications [9].

## 4. Conclusion

Safe SHD although technically challenging has progressively assumed a role in the treatment of Pipkin fractures. Whenever possible, these fractures should be stabilized in order to reduce the risk of early articular degenerative changes. The technique described by Ganz et al. has proven to be an effective and safe methodology compared to classical approaches, having the additional advantage to allow a 360° visualization of the femoral head and acetabulum.

## Figures and Tables

**Table 1:** Pipkin classification of femoral head fractures.

Type I	Fracture of the femoral head caudad to the fovea capitis femoris
Type II	Fracture of the femoral head cephalad to the fovea capitis femoris
Type III	Type I or II injury associated with fracture of the femoral neck
Type IV	Type I or II injury associated with fracture of the acetabular rim

## Additional Points

*Highlights*. Pipkin fractures are rare high-energy injuries with a high rate of complications. Surgical hip dislocation is a safe approach for internal fixation of Pipkin fractures. Surgical hip dislocation allows full acetabulum and femoral head access. Avascular necrosis or posttraumatic arthritis is the most common complication.
